# Swearing as a Response to Pain: Assessing Hypoalgesic Effects of Novel “Swear” Words

**DOI:** 10.3389/fpsyg.2020.00723

**Published:** 2020-04-30

**Authors:** Richard Stephens, Olly Robertson

**Affiliations:** ^1^School of Psychology, Keele University, Keele, United Kingdom; ^2^School of Psychology, Keele University, Keele, United Kingdom; ^3^Department of Experimental Psychology, University of Oxford, Oxford, United Kingdom

**Keywords:** swearing, cold-pressor, pain threshold, pain tolerance, emotion, humor, distraction

## Abstract

Previous research showing that swearing alleviates pain is extended by addressing emotion arousal and distraction as possible mechanisms. We assessed the effects of a conventional swear word (“fuck”) and two new “swear” words identified as both emotion-arousing and distracting: “fouch” and “twizpipe.” A mixed sex group of participants (*N* = 92) completed a repeated measures experimental design augmented by mediation analysis. The independent variable was repeating one of four different words: “fuck” vs. “fouch” vs. “twizpipe” vs. a neutral word. The dependent variables were emotion rating, humor rating, distraction rating, cold pressor pain threshold, cold pressor pain tolerance, pain perception score, and change from resting heart rate. Mediation analyses were conducted for emotion, humor, and distraction ratings. For conventional swearing (“fuck”), confirmatory analyses found a 32% increase in pain threshold and a 33% increase in pain tolerance, accompanied by increased ratings for emotion, humor, and distraction, relative to the neutral word condition. The new “swear” words, “fouch” and “twizpipe,” were rated as more emotional and humorous than the neutral word but did not affect pain threshold or tolerance. Changes in heart rate and pain perception were absent. Our data replicate previous findings that repeating a swear word at a steady pace and volume benefits pain tolerance, extending this finding to pain threshold. Mediation analyses did not identify a pathway via which such effects manifest. Distraction appears to be of little importance but emotion arousal is worthy of future study.

## Introduction

Swearing, defined as the use of taboo language conveying connotative information (Jay and Janschewitz, 2008), is a near-universal feature of language (van Lancker and Cummings, 1999). Research has shown that repeating a swear word can be an effective way of increasing tolerance for the physical pain of an ice water challenge (Stephens et al., 2009; Stephens and Umland, 2011; Robertson et al., 2017) and the social pain associated with ostracism (Philipp and Lombardo, 2017).

In explaining how swearing brings about these pain reducing effects, one theory posits that swearing brings about a stress-induced analgesia (Stephens and Umland, 2011; Philipp and Lombardo, 2017) via increased autonomic arousal. Consistent with this theory, several studies have shown that swearing provokes an autonomic response, assessed via increased heart rate (Stephens et al., 2009; Stephens and Umland, 2011) and increased skin conductance (LaBar and Phelps, 1998; Jay et al., 2008; Bowers and Pleydell-Pearce, 2011). It is the emotion-provoking aspect of swearing that is thought to underlie this increase in autonomic arousal (Stephens and Allsop, 2012).

It would be of theoretical interest to further assess the importance of emotional arousal as a means by which swearing brings about pain relief. A novel way to assess this would be to test whether a newly made-up “swear” word, chosen because it has potential to elicit an emotional response, produces similar pain reducing effects as swearing.

An alternative theory explaining how swearing brings about pain reducing effects is via attention modulation (Wiech et al., 2008). It is established within the framework of the descending pain inhibitory system that cognitive processes, including distracting attention away from a pain stimulus, can reduce perceived pain (Edwards et al., 2009). The precise mechanism appears to be a combination of inhibiting sensory and emotional brain regions, while at the same time acting in an excitatory capacity on the periaqueductal gray region of the brain where endogenous opioids such as endorphins are produced (Sims-Williams et al., 2017).

One property of swearing that may usefully distract one’s attention from pain is if the word is perceived as humorous or novel. That swearing can be perceived as funny has been shown by Engelthaler and Hills (2018), who had 821 participants rate 5000 English words for humor. The word “fuck” was rated in the top 1% of funniest words. Similarly, swearing is perceived as a novel unit of language, evidenced by findings estimating that swear words make up less than 1% of all speech (Jay, 2009). Given that human attention appears biased toward detecting stimuli that occur less frequently over those occurring more frequently (Horstmann and Herwig, 2016), it would be of theoretical interest to further assess the importance of attention modulation as a means by which swearing brings about pain relief. A novel way to assess this would be to test whether a newly made-up “swear” word, chosen because it has potential to elicit distraction through humor, novelty, or some other aspect, produces similar pain reducing effects as swearing.

The aim of this research study was to generate two new “swear” words, defined as non-pre-existing words that can be used in place of swear words, and to assess the pain-relieving effects of repeating these new words in the context of a cold pressor (ice water) pain challenge. The study provided an opportunity to explore some of the properties of swear words that underlie their psychological effects. Including new “swear” words enabled isolation of some of the properties of swear words in the absence of learned associations that true swear words have been theorized to possess (Jay, 2009).

The new “swear” were generated by an agency working for Nurofen. They were selected for the experiment by a panel consisting of the lead author, a lexicographer, an independent scientist with expertise in swearing, and two lay members. The selection process for the new “swear” words is described later. The cold pressor experiment included the two new words, “fouch” and “twizpipe,” alongside a conventional swear word, “fuck,” included partly as a research replication paradigm. There was also a neutral word control condition to provide a reference against which to assess the effects of the conventional and new swear words. This was a word to describe a table in line with previous similar studies (e.g., Stephens and Umland, 2011). Key aspects of this study were pre-registered on aspredicted.org (#21777) (see **Supplementary Materials**). Hypotheses (i) to (vii) were included on the pre-registration document and should be considered confirmatory, although please note that, in error, we specified one-way unrelated ANOVAs rather than one-way related ANOVAs. Hypothesis (viii) was not included on the pre-registration document and consequently should be considered exploratory.

It was hypothesized: (i) that emotion ratings would be greater for “fouch” vs. neutral word; (ii) that humor and distraction ratings would be greater for “twizpipe” vs. neutral word; (iii) that emotion, humor, and distraction ratings would be greater for “fuck” vs. neutral word; (iv) that cold pressor pain onset latency (pain threshold) would be increased for “fuck,” “fouch,” and “twizpipe” vs. neutral word; (v) that cold pressor pain tolerance latency would be increased for “fuck,” “fouch” and “twizpipe” vs. neutral word; (vi) that pain perception would be decreased for “fuck,” “fouch” and “twizpipe” vs. neutral word; (vii) that change from resting heart rate would be increased for “fuck” and “fouch” vs. neutral word; and (viii) that the effects of swearing on pain tolerance would be mediated by one or more of the emotion rating, humor rating, or distraction rating scores.

## Materials and Methods

### Participants

We recruited 102 male and female adults from around Keele University who were offered payment of £8 cash (which two participants declined). Exclusion criteria included first language other than English, having a chronic pain condition, a heart condition, circulatory problems, high or low blood pressure, diabetes, epilepsy, Raynaud’s syndrome, having taken analgesic medications within 12 h, recent serious injury, and history of fainting in the last 12 months. Ten participants were excluded at the data analysis stage due to: a first language other than English (*n* = 4), missing cold pressor data due to experimenter error (*n* = 3), and withdrawal without completing the protocol (*n* = 3). The remaining sample of 92 individuals (59 females; 32 males; 1 preferred not to say) with mean age 27.8 years (*SD* = 9.0) was put forward for analysis (see **Supplementary Materials**). The sample size was guided by a power calculation based on previous research on the hypoalgesic effects of swearing that yielded medium to large effect sizes (*dz* range: 0.62–1.12; Stephens et al., 2009; Stephens and Umland, 2011; Stephens and Allsop, 2012). Based on a conservatively estimated small to medium effect size of *dz* = 0.30, we calculated that 90 participants would be required for a within-subjects comparison of an experimental word versus a control word, with alpha set at 0.05 and power set at 80%. This study was carried out in accordance with the ethical recommendations of the British Psychological Society. The protocol was approved by the Keele University Psychology Faculty Research Ethics Committee. All subjects gave written informed consent in accordance with the Declaration of Helsinki.

### Materials

#### Cold Pressor

A 185 mm × 335 mm × 215 mm (height) Grant^TM^ water bath with circulating pump was maintained at 3–5°C by adding crushed ice between trials. This size of bath enabled the full open hand to be immersed in water of a depth of approximately 120 mm. There was a 3-minute maximum time limit, assessed via a handheld stopwatch, which was not explicitly communicated to the participants; 20 participants reached this maximum on at least one trial, while 10 participants reached it on all four trials. A container of water at room temperature was present. The water in the ice water and room temperature baths was refreshed daily.

#### Heart Rate

This was assessed throughout the procedure using a BIOPAC Systems Inc., MP36 four-channel data acquisition unit in combination with BIOPAC Student Lab 4.1 software. Pre-gelled disposable electrodes (type EL503) were placed at the medial surface of the right leg just above the ankle bone (ground), at the medial surface of left leg just above the ankle bone (live), and at the anterior forearm just above the wrist on the same side of arm as the palm of the preferred hand (neutral). Digital markers were dropped on to the heart rate recordings at the start and end of the 30 s resting heart rate measurement period and at the start and end of each ice water immersion. Mean heart rate (beats per minute) was calculated within the BSL Student Lab software and checked visually. No filters were applied; where the data were too noisy for automatic detection, manual peak-to-peak counts were carried out for the first 30 s of the epoch and mean heart rate was calculated for this period. Five epochs of heart rate were used in analyses: resting heart rate, and heart rate during the four experimental cold pressor immersions. Change from resting heart rate data are reported to maintain consistency with previous studies assessing effects of swearing on pain perception. The data reported are considered sustained measures of heart rate based on the criteria suggested by Jennings et al. (1981).

#### Pain Threshold and Pain Tolerance

Pain threshold was measured in seconds. A digital marker was dropped on to the heart rate recording when the participant indicated that they perceived pain. Pain tolerance was measured in seconds; tolerance was assessed by the total time the participant submerged their hand in the ice-cold water.

#### Manipulating Vocalizations

Four manipulations of vocalization were employed: swearing (“fuck”); two new “swear” words; and a neutral word, which was a word chosen by the participants that describes a table (e.g., “solid”). The new “swear” words were selected by a panel comprising the lead author as chair, along with Dr. Emma Byrne, a freelance science writer who has written a popular science book entitled “Why Swearing is Good for You” (Byrne, 2017), Jonathon Green, a lexicographer and author of several slang dictionaries (Green, 2010, 2008), and two lay members of the public with no language qualifications beyond A-levels. During a 2-hour meeting a long list of 60 candidate new “swear” words, created by an advertising agency (as described earlier), was considered. Unsuitable words were discarded until two remained. A steer was provided that one of the new “swear” words should carry emotional resonance, while the other should offer distraction, possibly via humor. Discussion ran to members of the panel shouting the words from outside the room, as well as discussing when or how they might use the words. A consensus was eventually reached in nominating “fouch” as the new “swear” word with potential to invoke emotion, and “twizpipe” as the new “swear” word with potential to invoke distraction via humor. For each cold pressor trial undertaken, the participant was asked to repeat the pertinent word (“fuck,” “fouch,” “twizpipe,” or neutral word) at a normal speech volume and a steady pace, once every 3 s.

#### Perceived Pain Scale

Developed by Borg (1998), this single item questionnaire asks participants to rate how strongly they perceived the pain of a stimulus (here the ice water immersion) on a scale from 0, anchored “Nothing at all,” to 12, anchored “Absolute maximum.” Additional anchors are at 0.5: “Extremely weak”; 1: “Very weak”; 2: “Weak”; 3: “Moderate”; 5: “Strong”; and 7: “Very strong.” Possible scores range from 0 to 12, with a higher score indicative of a greater level of perceived pain. This scale has been previously used to assess effects of swearing on pain perception (Stephens et al., 2009; Stephens and Umland, 2011).

#### Pain Catastrophizing Questionnaire

Developed by Sullivan et al. (1995), this 13-item questionnaire (e.g., “I think the pain will be awful”) is answered on a 5-point Likert scale anchored from “Not at all,” scored 0, to “All the time,” scored 4. Possible scores range from 0 to 46, with a higher score indicative of a greater level of pain catastrophizing. In our sample this questionnaire showed good reliability, Cronbach’s α = 0.903, *N* = 92.

#### Fear of Pain Questionnaire Version 3

Developed by McNeill and Rainwater (1998), this 30-item questionnaire asks participants to rate how fearful they find each example (e.g., “breaking your leg”) on a 5-point Likert scale anchored from “Not at all,” scored 1, to “Extreme,” scored 5. Possible scores range from 30 to 150, with a higher score indicative of a greater level of fear of pain. In our sample this questionnaire showed good reliability, Cronbach’s α = 0.913, *N* = 91. Please note that one participant accidentally omitted to complete the Fear of Pain Questionnaire.

#### Word Ratings

Participants rated each word after the trial in which they used it on three dimensions: emotion (“Repeating the word made me feel an emotion along the lines of excitement, anger or fear”); humor (“Repeating the word was funny/humorous”); and distraction (“Repeating the word distracted me from thinking about other things”). Ratings were made on Visual Analog Scales (VAS), each consisting of a 100 mm horizontal line anchored at its left side with “Not at all” and at its right side “A lot.” These were scored by measuring the distance of the mark from the left-hand end of the VAS (mm). The possible range of scores was 0–100 with a higher score indicating greater ratings of emotion, humor, and distraction. Although not specifically validated to assess these constructs, using a VAS has been found to be a reliable and valid psychometric method in the context of quality of life (DeBoer et al., 2004).

### Design

A one-way repeated measures experimental design was applied with four conditions defined by repeating each of the following word-types during cold pressor hand immersion: conventional swear word (“fuck”); new swear word#1 chosen because it promotes emotional resonance (“fouch”); New swear word#2 chosen because it is distracting/humorous (“twizpipe”); and neutral word control condition (a word to describe a table). A cold pressor ice-water hand immersion task was utilized. The dependent variables were: pain onset latency (time from submersion to feeling of pain); pain tolerance latency (total submersion time); pain perception (Borg rating scale completed after each immersion); heart rate (gathered using BIOPAC); and word ratings (each word was rated for emotion, humor, and distraction). Scores on the Pain Catastrophizing Questionnaire and Fear of Pain Questionnaire Version 3 were compiled for the purposes of descriptive data. Condition order was randomized to counter order effects using the Microsoft Excel random number generation command “ = RAND().”

### Procedure

Student research assistants were engaged for the data collection under supervision of the authors. In order not to give away the aim of the research to participants, recruitment materials referred to the study with the title “Psychological effects of vocal expressions, including swearing, while immersing the hand in ice water.” Participants attended the research laboratory individually. On arrival they were asked to read an “Information for Participants” sheet, offered the opportunity ask any questions and, when satisfied, asked to sign a consent form as a verifiable record of informed consent. Participants were asked to nominate a word that can describe a table, which was to be the neutral word control condition. Next, they were fitted with adhesive electrodes in three locations: at the wrist of the preferred arm, and the inner part of each ankle. To record resting heart rate, participants sat quietly in a chair for 5.5 min, with resting heart rate recorded as the mean heart rate for the final 30 s of this period. Participants were then asked to complete the Pain Catastrophizing Questionnaire and the Fear of Pain Questionnaire.

After this, participants were asked to immerse their non-preferred hand in the room temperature bath for 3 min. This enabled a standardized starting temperature for the ice water immersions. The instruction for the ice water immersion were: “In a moment I would like you to fully immerse your non-preferred hand into this ice water bath. While it is submerged please repeat the word [INSERT AS APPROPRIATE] at normal speech volume and a steady pace, once every 3 s. While you have your hand in the water, I would like you to do TWO more things. First, please tell me when it becomes painful, but don’t take your hand out yet unless you have to. Second, please try and keep your hand in the water for longer, taking it out when the pain becomes unbearable.” Timing began when the hand was fully immersed and stopped when the hand was fully removed from the water.

**FIGURE 1:**
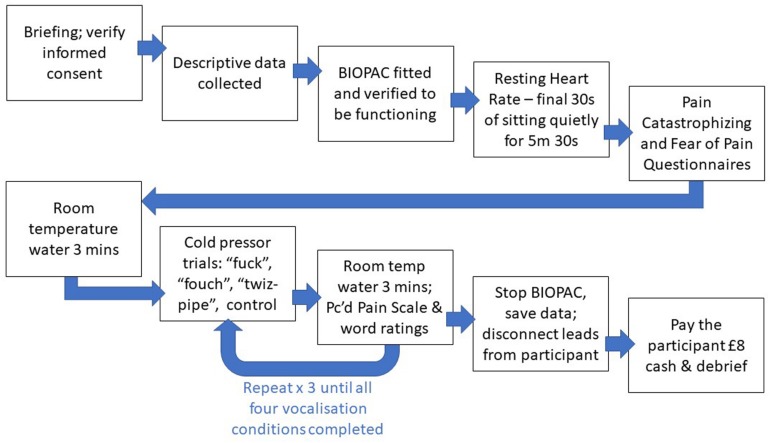
Diagram of the study procedure.

Immediately after each cold pressor submersion, participants immersed the non-preferred hand in the room temperature bath for 3 min prior to the next cold pressor trial. The Perceived Pain Scale and word ratings were administered at this juncture. After all four trials were complete a paper towel was made available, and participants were thanked and debriefed. Please see Figure 1.

## Results

Descriptive data appear in Table 1. Analyses were performed using SPSS v24. As described in the pre-registration document, all dependent variables were checked for normality. Outliers were defined as values that were more than 1.5 times above or below the interquartile range, which is the default for SPSS box and whisker plots. It was not possible to eliminate outliers without removing an excessive number of cases (more than ten) and therefore some variables were Winsorised, following the method of Aguinis et al. (2013), as shown in Table 1. The Winsorization percentile column of Table 1 shows that the number of outliers varied across different variables, from 0 to 35%. This reflects that the number of outliers varied from 0–16 cases. Following Winsorisation, skewness and kurtosis coefficients were checked for all variables and found to be within the range: −1.302 to 1.123.

**TABLE 1:** Descriptive data.

	**Mean**	**SD**	**95%CI lower**	**95%CI upper**	**Winsorization percentile**
Pain Catastrophizing score	22.33	10.42	20.17	24.48	–
Fear of Pain Questionnaire score	88.19	17.6	84.52	91.85	–
**Waterbath temperature (°C)**					
Fuck	3.91	0.51	3.8	4.02	–
Fouch	3.98	0.5	3.88	4.09	–
Twizpipe	3.92	0.53	3.81	4.03	–
Neutral	3.97	0.5	3.86	4.07	–
**Pain threshold (s)**					
Fuck	35.77*	23.88	33.18	38.36	85th
Fouch	28.23	17.09	25.64	30.82	87th
Twizpipe	25.45	15.59	22.86	28.04	82nd
Neutral	27.2	18.24	24.61	29.79	77th
**Pain tolerance (s)**					
Fuck	74.28*	57.53	69.42	79.14	–
Fouch	58.44	41.32	53.59	63.3	72nd
Twizpipe	52.47	33.41	47.62	57.33	70th
Neutral	55.87	38.48	51.01	60.73	65th
**Perceived Pain rating scale score**					
Fuck	4.95	2.32	4.67	5.23	–
Fouch	4.99	2.07	4.71	5.27	–
Twizpipe	5.37	2.23	5.09	5.66	–
Neutral	5.17	2.3	4.89	5.46	–
**Change from resting heart rate (bpm)**					
Fuck	15.94	11.71	14.74	17.14	98th
Fouch	14.33	9.39	13.13	15.53	98th
Twizpipe	14.71	8.47	13.51	15.91	93rd
Neutral	15.21	10.04	14.01	16.41	93rd
**Emotion rating**					
Fuck	35.30*	26.33	32.36	38.24	–
Fouch	22.49*	21.27	19.55	25.43	98th
Twizpipe	21.97*	22.52	19.03	24.91	89th
Neutral	17.41	19.26	14.47	20.35	70th
**Humor rating**					
Fuck	48.85*	29	44.31	53.38	–
Fouch	41.48*	27.69	36.94	46.01	–
Twizpipe	50.98*	30.35	46.44	55.51	–
Neutral	30.5	29.93	25.96	35.04	–
**Distraction rating**					
Fuck	60.39*	22.59	56.37	64.41	–
Fouch	46.39	25.91	42.37	50.41	–
Twizpipe	45.74	27.07	41.72	49.76	–
Neutral	42.27	27.07	38.25	46.29	–

*Comparison versus neutral word was significant, *p* < 0.05.

### Manipulation Checks

**FIGURE 2:**
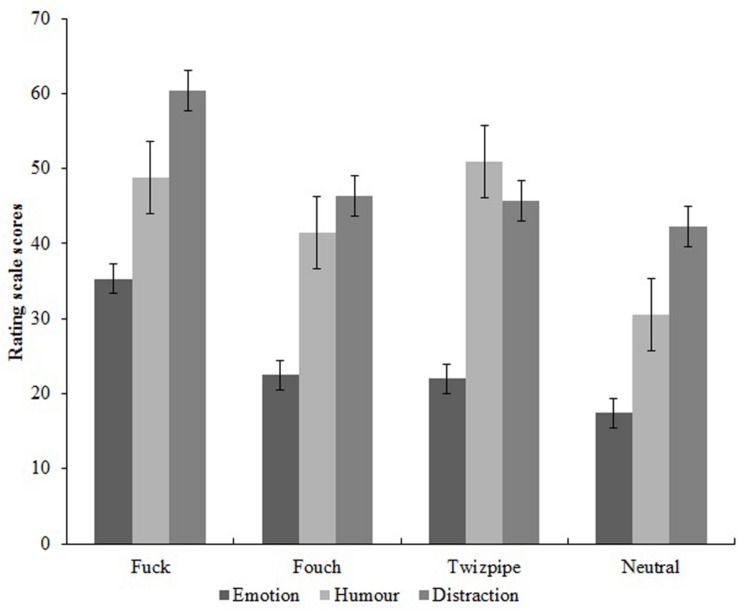
Effect of experimental condition (word repeated) on the emotion, humor, and distraction rating scale scores.

A series of one-way repeated measures ANOVAs were carried out for the independent variable, Word, with the levels, “fuck” vs. “fouch” vs. “twizpipe” vs. neutral word, and for the dependent variables, emotion rating, humor rating, and distraction rating. Where Mauchley’s test indicated significant departures from sphericity, Huynh-Feldt corrections are reported. These ANOVAs found significant differences across the means for emotion rating, *F*(2.855,259.761) = 27.821, *MSe* = 205.330, *p* < 0.001, η*p*^2^ = 0.234, for humor rating, *F*(3,273) = 16.106 *MSe* = 488.200, *p* < 0.001, η*p*^2^ = 0.150, and for distraction rating, *F*(3,273) = 15.346 *MSe* = 383.886, *p* < 0.001, η*p*^2^ = 0.144. These are depicted in Figure 2.

Pairwise comparisons for emotion rating showed that fuck, *F*(1,259.8) = 71.701, *p* < 0.001, fouch, *F*(1,259.8) = 5.781, *p* = 0.017, and twizpipe, *F*(1,259.8) = 4.658, *p* = 0.032, gained significantly higher ratings than the neutral word. Pairwise comparisons for humor rating again showed that fuck, *F*(1,273) = 31.720, *p* < 0.001, fouch, *F*(1,273) = 11.356, *p* = 0.001, and twizpipe, *F*(1,273) = 39.513, *p* < 0.001, gained significantly higher ratings than the neutral word. However, for distraction rating, pairwise comparisons showed that fuck was rated significantly higher than the neutral word, *F*(1,273) = 39.343, *p* < 0.001, but that neither fouch nor twizpipe showed any difference compared with the neutral word, *F*(1,273) < 1.0.

### Pain Outcomes

**FIGURE 3:**
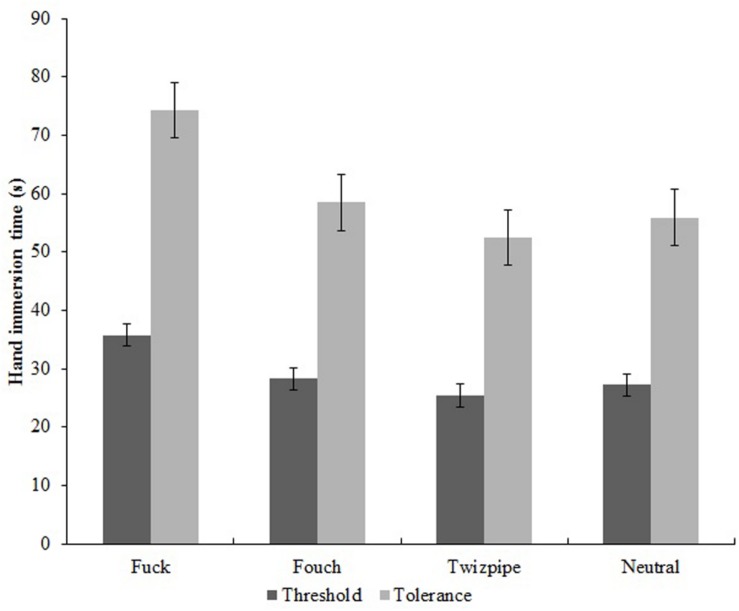
Effect of experimental condition (word repeated) on pain onset latency (threshold) and pain tolerance latency (tolerance).

A series of one-way repeated measures ANOVAs were carried out for the independent variable, Word, with the levels, “fuck” vs. “fouch” vs. “twizpipe” vs. neutral word, and for the dependent variables, cold pressor pain threshold (pain onset latency), cold pressor pain tolerance latency, pain perception score, and change from resting heart rate. Again, where Mauchley’s test indicated significant departures from sphericity, Huynh-Feldt corrections are reported. These ANOVAs found significant differences across the Word types for pain onset, *F*(2.779,216.789) = 11.123, *MSe* = 158.739, *p* < 0.001, η*p*^2^ = 0.125 and for pain tolerance, *F*(2.432,221.285) = 18.917, *MSe* = 559.518, *p* < 0.001, η*p*^2^ = 0.172, but not for pain perception, *F*(3,246) = 1.651, *MSe* = 1.893, *p* = 0.178, η*p*^2^ = 0.020, nor for change from resting heart rate, *F*(2.806,244.129) = 1.336, *MSe* = 34.159, *p* = 0.263, η*p*^2^ = 0.015. Effects for cold pressor pain onset latency (threshold) and pain tolerance latency are depicted in Figure 3.

Pairwise comparisons for pain onset latency showed that these were longer for fuck compared with the neutral word, *F*(1,261.8) = 21.283, *p* < 0.001. However, pain onset latencies for fouch, *F*(1,261.8) < 1.0, and twizpipe, *F*(1,261.8) < 1.0, were not different to that for the neutral word. A similar pattern was present for pain tolerance, with longer pain tolerance latencies for fuck compared with the neutral word, *F*(1,221.3) = 27.865, *p* < 0.001, but again, pain tolerance latencies for fouch, *F*(1,221.3) < 1.0, and twizpipe, *F*(1,221.3) < 1.0, were not different to those for the neutral word.

A peer reviewer suggested running equivalence tests assessing whether the null effects for fouch and twizpipe were less than the smallest effect size of interest. These analyses were not specified in the pre-registration document and should be considered exploratory. We used the TOST procedure (Lakens, 2017), setting the smallest effect size of interest at *dz* = 0.30, which was the conservatively small estimate of expected effect size entered into the power calculation upon which sample size was determined. For pain threshold, the TOST procedure indicated that the observed effect size for fouch (*dz* = 0.07) was significantly within the equivalent bounds of *dz* = −0.3 and *dz* = 0.3, (or in raw scores: −4.37 and 4.37), *t*(91) = −2.2, *p* = 0.015. The same was the case for twizpipe (observed effect size *dz* = −0.12; equivalent bound raw scores: −4.24 and 4.24), *t*(91) = 1.69, *p* = 0.047. For pain tolerance, the observed effect size for fouch (*dz* = 0.09) was also significantly within the equivalent bounds of *dz* = −0.3 and *dz* = 0.3, (raw scores: −8.14 and 8.14), *t*(91) = −1.97, *p* = 0.026. The observed effect size for twizpipe (*dz* = −0.16) was not significantly within the upper and lower equivalent bounds of *dz* = −0.3 and *dz* = 0.3, (or in raw scores: −6.35 and 6.35), *t*(91) = 1.34, *p* = 0.092. However, while this default two-tailed TOST procedure was inconclusive, the effect of most interest was the one-tailed upper bound test assessing whether twizpipe might produce pain relieving effects above *dz* = 0.3. This upper bound TOST procedure found that twizpipe was within the *dz* = 0.3 limit, *t*(91) = 4.42, *p* < 0.001.

### Mediation

In exploratory analyses not specified in the pre-registration document, a series of tests of mediation were conducted using the MEMOREv2.0 SPSS MACRO command for within-subjects designs (Montoya and Hayes, 2017). The aim was to assess whether the observed effect of swearing (“fuck” vs. neutral word) on pain tolerance was mediated by emotion, humor, or distraction, as measured using the rating scales. The default setting of applying 5,000 bootstrapped samples in the estimation of 95% CI around the indirect effect model was applied.

While swearing predicted both pain tolerance, β = 18.411, 95% CI = 11.216:25.605, and emotion, β = 17.891, 95% CI = 13.143:22.640, emotion did not predict pain tolerance, β = 0.266, 95% CI = −0.070:0.602. This analysis further found that the indirect effect of swearing on pain tolerance via emotion was not significant, β = 4.767, 95% CI = −0.093:4.282. This latter effect is an estimate of the extent to which swearing affects pain tolerance via the emotion rating scale score. Swearing also predicted humor ratings, β = 18.349, 95% CI = 11.698:24.998, but humor ratings did not predict pain tolerance, β = −0.035, 95% CI = −0.263:0.192. The indirect effect of swearing on pain tolerance via humor ratings was not significant, β = −0.645, 95% CI = −5.086:3.303. Finally, swearing was confirmed as predicting distraction ratings, β = 18.120, 95% CI = 12.525:23.714, but distraction ratings did not predict pain tolerance, β = −0.064, 95% CI = −0.340:0.212. The indirect effect of swearing on pain tolerance via distraction ratings was not significant, β = −1.164, 95% CI = −5.031:3.852.

## Discussion

This study contributes to the psychology literature on swearing in the context of pain (Stephens et al., 2009; Stephens and Umland, 2011; Philipp and Lombardo, 2017; Robertson et al., 2017) as the first attempt to create new “swear” words and assess some of their psychological properties. Our experiment assessed the effects of repeating three different words – a conventional swear word (“fuck”) and two new “swear” words (“fouch” and “twizpipe”) - on pain perception and tolerance, compared with a neutral word control condition (a word to describe a table). We ran a well-powered experiment with a sample consisting of 92 native English speakers. We used an ice-cold water hand immersion task known as the cold pressor procedure. This provides a controlled stimulus that is painful but not harmful and yields scores for pain threshold (time at which pain is reported) and pain tolerance (time at which the hand is removed). We also recorded heart rate as well as ratings of pain perception, emotion, humor, and distraction. The order in which participants completed the conditions (“fuck,” “fouch,” “twizpipe,” and neutral word) was randomized to guard against order effects. Pain Catastrophizing and Fear of Pain scores were gathered to help understand sample characteristics. The scores were similar to our previous data (Stephens and Umland, 2011) in which the overall mean score for Pain Catastrophizing was 25.30 (*SD* = 9.64) and for Fear of Pain was 87.45 (*SD* = 16.43). This indicates that our sample may be considered typical for these variables and, as such, that these variables are unlikely to have unduly influenced the pain outcomes.

Hypotheses (i) to (iii) were put forward as manipulation checks to ensure that the made-up “swear” words had the desired properties in terms of the emotion, humor, and distraction ratings. Hypothesis (i) that emotion ratings would be greater for “fouch” vs. neutral word was supported, and hypothesis (ii) that humor and distraction ratings would be greater for “twizpipe” vs. neutral word was partially supported in that the humor rating was greater for “twizpipe.” Interestingly, both made-up “swear” words showed higher ratings for emotion and humor compared with the neutral word. Hypothesis (iii) that emotion, humor, and distraction ratings would be greater for “fuck” vs. neutral word was supported. Our tests of hypotheses (i) to (iii) demonstrate that our manipulation of creating new “swear” words was successful in that “fouch” and “twizpipe” were able to evoke some of the properties of swearing, in terms of emotion rating and humor. This was not the case for distraction, however, since only “fuck” was found to have a raised distraction rating compared with the neutral word. Given that both new “swear” words had demonstrated potential to influence pain perception via increased emotion ratings and/or distracting a person from the pain via increased humor ratings, it seemed appropriate to continue with the analyses and test whether the new “swear” words had any effect on the pain outcomes. We also note that “fuck” was rated as humorous in this context, consistent with the findings of Engelthaler and Hills (2018), who found the word “fuck” was rated in the top 1% of funniest words when 5000 English words were presented one at a time.

Hypotheses (iv) to (vii) were put forward as tests of whether the conventional swear word and the new “swear” words would show hypoalgesic effects and associated changes in heart rate, as found previously. Hypothesis (iv), that cold pressor pain onset latency (pain threshold) would be increased for “fuck,” “fouch,” and “twizpipe” vs. neutral word, was supported for “fuck” but not for “fouch” or “twizpipe.” Hypothesis (v), that cold pressor pain tolerance latency would be increased for “fuck,” “fouch,” and “twizpipe” vs. neutral word, was also supported for “fuck” but not for “fouch” or “twizpipe”. Together, these findings extend previous research on swearing and pain by replicating, in a pre-registered study, the beneficial effect of swearing on pain tolerance and showing that swearing has an additional beneficial effect on pain threshold (onset latency), a behavioral pain measure that has not previously been assessed.

Regarding the new “swear” words, our confirmatory analyses showed no beneficial effects for pain threshold and tolerance. On the suggestion of a peer reviewer, we ran exploratory equivalence tests assessing whether the effect sizes for these words were within a range considered to be negligible. These analyses confirmed the absence of a beneficial effect for pain threshold and tolerance beyond a smallest effect size of interest based on the conservatively small estimate of *dz* = 0.3 entered into the power calculation. That these new “swear” words had no effect on pain threshold and tolerance is not altogether surprising. While it is not properly understood how swear words gain their power, it has been suggested that swearing is learned during childhood and that aversive classical conditioning contributes to the emotionally arousing aspects of swear word use (Jay, 2009; Tomash and Reed, 2013). This suggests that how and when we learn conventional swear words is an important aspect of how they function. Clearly, the new “swear” words utilized in the present study were not learned during childhood and so there was no possibility that this aspect could have had an influence. On the other hand, “fouch” and “twizpipe” were chosen because they had potential to mirror some properties of conventional swearing. Like the swear word, these words were rated as more emotion-evoking and humorous than the neutral word control condition. Nevertheless, these properties did not facilitate pain alleviation effects, suggesting that surface properties of swear words (such as how they sound) do not explain the hypoalgesic effects of swearing. An overall absence of pain alleviation effects for the new “swear” words in the present study would be expected based on Jay’s (2009) childhood aversive classical conditioning theory. There is little evidence for this theory other than a low powered experiment (*N* = 26) finding that participants reporting a higher frequency of punishment for swearing as children showed an increased skin conductance response when reading swear words, compared with participants reporting a lower frequency of punishment for swearing (Tomash and Reed, 2013). To investigate this theory further, future research should aim to verify the frequency with which such aversive classical conditioning events occur in childhood and assess the relationship between prior punishment for swearing and autonomic arousal in an adequately powered design.

Hypothesis (vi), that pain perception would be decreased for “fuck,” “fouch,” and “twizpipe” vs. neutral word, was not supported. We should not be surprised at the lack of differences for pain perception as this may indicate that participants base behavioral decisions of reporting pain onset and removing the hand on similar perceived pain levels, albeit levels that have been modified by repeating a swear word. On that basis we suggest that measuring subjective pain perception is of limited usefulness in future studies assessing hypoalgesic effects of swearing where behavioral measures such as the cold pressor procedure are employed.

Hypothesis (vii), that change from resting heart rate would be increased for “fuck” and “fouch” vs. neutral word, was not supported. The lack of heart rate differences across conditions is at odds with previous studies which have shown elevated heart rate for swearing versus a neutral word (Stephens et al., 2009; Stephens and Umland, 2011). This may be due to the design of the present study in which participants completed four consecutive word repetition/cold pressor immersion conditions rather than two, as previously. Repeated presentations of similar tasks, as well as repeated exposure to aversive stimuli, have been found to result in blunted cardiovascular stress reactivity (Hughes et al., 2018). Blunted cardiovascular stress reactivity refers to the reduction in cardiovascular response to acute physiological or psychological stress (Brindle et al., 2017). It seems reasonable to suggest that repeated exposure to cold pressor-mediated acute pain may have induced cardiovascular blunting.

In the absence of clear autonomic responses to swearing, we assessed the exploratory hypothesis (viii) that the effects of swearing on pain tolerance would be mediated by one or more psychological variables, in the form of the emotion, humor, or distraction rating scores. However, none of the ratings showed evidence of mediation, with 95% confidence intervals for humor and distraction being approximately symmetrically balanced across the origin. The latter effect is of interest because swearing in the context of pain is often characterized as a deliberate strategy for distraction, and distraction is recognized as being an effective psychological means of influencing descending pain inhibitory pathways (Edwards et al., 2009). While swearing was rated as distracting (more so than the other words) the level of distraction was not related to the pain alleviation effects. Thus, based on our evidence, distraction may not be important in explaining how swearing produces hypoalgesic effects. The analysis assessing whether emotion ratings mediate the effect of swearing on extending pain tolerance also showed no effect, although here the 95% confidence interval only narrowly crossed the origin. While offering no evidential support for a mediation effect, further study assessing mediation of hypoalgesic effects of swearing via emotional arousal, in the absence of changes in heart rate, might fruitfully demonstrate this as a viable mechanism. Such an effect would be in keeping with previous research finding pain relieving effects of emotional arousal (Stephens and Allsop, 2012).

However, there is a caveat to this. At the study outset we theorized that swearing may increase emotional arousal without specifying the valence of that arousal. During peer review we were directed to literature linking emotion elicitation and pain modulation, and in particular, research by Lefebvre and Jensen (2019) who report that inducing a state of negative affect by asking participants to recall a time when they experienced a high degree of worry led to increased ratings of pain from pressure applied to the finger, relative to baseline. In addition, the same study found that inducing a state of positive affect by asking participants to recall a happy memory led to decreased ratings of pain. It is apparent that emotional modulation of pain can be explained by the two-factor behavioral inhibition system-behavioral activation system (BIS-BAS) model of pain (Jensen et al., 2016). According to the BIS-BAS model, negative affect contributes toward pain-related avoidance behaviors and associated negative cognitions, thereby increasing the subjective experience of pain. Conversely, positive affect contributes toward approach behaviors and positive cognitions, thus decreasing the subjective experience of pain. One limitation of the present study is that the measure of emotion elicitation was not valenced. This may explain why emotion was not shown to be a mediating variable in the link between swearing and hypoalgesia. Future research should assess both positive and negative emotion arousal due to swearing.

A further limitation might have been that participants did not consider themselves to be swearing when repeating the novel “swear” words. This remains unknown as we did not carry out a manipulation check asking participants whether they considered using these words was swearing. On the other hand, the novel “swear” words were selected by a panel of experts and laypeople briefed to choose words that could be used in similar ways to swear words, and which shared properties of swear words including emotional resonance and humor potential. It is also worth noting that “Fouch” begins with a fricative, defined as a sound created by forcing air through a narrow channel (here the lower teeth and upper lip) which some have associated with swearing, although other contest such a link (Stack Exchange, 2014).

Additionally, maintaining the ice water temperature in the range 3–5°C might be considered too wide a variation, such that the physical intensity of the pain stimulus was not consistent across participants. In mitigation there was no systematic variation of the temperature across the four word conditions. As shown in Table 1, the starting temperatures for each immersion were fairly consistent, with means ranging from 3.91 to 3.98 (SDs 0.50 to 0.53). This indicates that approximately 65% of immersions had starting temperatures within a 1°C range of 3.5–4.5°. Therefore, variation in temperature is unlikely to have biased the results.

A final limitation is that participants may have guessed the aims of the study and consequently demand characteristics may have influenced the results. In advertising the study as “psychological effects of vocal expressions, including swearing, while immersing the hand in ice water” we aimed to hide our predictions. Nevertheless, due to widespread media exposure for findings of previous studies conducted in the Keele Swear Lab we cannot rule out, nor quantify the extent to which, participant behavior was influenced by expectations of participants.

## Conclusion

This is the first study to find that new, made-up “swear” words do not have similar pain alleviation effects to regular swearing. While the new “swear” words were shown to be similar to swearing in terms of eliciting raised emotion and humor ratings, these words were not effective in alleviating pain onset or pain tolerance. On the other hand, our study is the first to show that swearing raises pain threshold (the time at which pain onset is reported following presentation of a painful stimulus, here immersing the hand in ice-water) building on previous findings showing that swearing raises pain tolerance (the time at which the hand is removed from the ice-water). It is also the first study to investigate mediation via distraction, finding no evidence that distraction is involved in the mechanism by which swearing brings about pain alleviation. Instead, our data suggest that swearing brings about its effect on pain alleviation via another route, possibly emotion arousal. However, emotion was not found to mediate the pain alleviation effects of swearing, so this remains a theoretical possibility rather than one that was evidenced.

## Author’s Note

A preprint of the first draft of this paper is available here: https://psyarxiv.com/cdvyf.

## Data Availability Statement

The pre-registration document and full datasets can be found in the Open Science Framework https://osf.io/fg2a9/.

## Ethics Statement

The studies involving human participants were reviewed and approved by Keele University Psychology Faculty Research Ethics Committee. The patients/participants provided their written informed consent to participate in this study.

## Author Contributions

RS and OR contributed to conception and design of the study. RS organized the database, performed the statistical analysis, and wrote the first draft of the manuscript. OR wrote sections of the manuscript. Both authors contributed to manuscript revision, read, and approved the submitted version.

## Conflict of Interest

The authors declare that the research was conducted in the absence of any commercial or financial relationships that could be construed as a potential conflict of interest.
